# High serum MMP-14 predicts worse survival in gastric cancer

**DOI:** 10.1371/journal.pone.0208800

**Published:** 2018-12-07

**Authors:** Aaro Kasurinen, Taina Tervahartiala, Alli Laitinen, Arto Kokkola, Timo Sorsa, Camilla Böckelman, Caj Haglund

**Affiliations:** 1 Research Programs Unit, Translational Cancer Biology, University of Helsinki, Helsinki, Finland; 2 Department of Oral and Maxillofacial Diseases, Helsinki University Hospital and Biomedicum Helsinki, Helsinki, Finland; 3 Department of Surgery, University of Helsinki and Helsinki University Hospital, Helsinki, Finland; 4 Department of Dental Medicine, Karolinska Institutet, Huddinge, Sweden; Peking University Cancer Hospital and Institute, CHINA

## Abstract

Matrix metalloproteinases (MMPs), endopeptidases with diverse biochemical functions, can promote cancer cell invasion and metastasis by degrading the extracellular matrix. A high matrix metalloproteinase-14 (MMP-14) expression in gastric cancer tissue has been associated with metastasis and poor prognosis. To further understand this association, we investigated serum MMP-14 as a biomarker in gastric cancer patients. The patient cohort consisted of 240 gastric adenocarcinoma patients who underwent surgery at Helsinki University Hospital, Finland, between 2000 and 2009. We determined the soluble MMP-14 serum levels using an enzyme-linked immunosorbent assay. We then calculated the associations between serum levels and clinicopathologic variables using the Mann-Whitney U-test or the Kruskal-Wallis test. We constructed survival curves using the Kaplan-Meier method and calculating the hazard ratios using the Cox proportional hazard model. We revealed a positive association between a high serum MMP-14 level and stages III–IV (p = 0.029), and between a high serum MMP-14 and distant metastasis (p = 0.022). Patients with a low serum MMP-14 had a 5-year disease-specific survival of 49.2% (95% confidence interval [CI] 45.5–52.9), whereas patients with a high serum MMP-14 had a 5-year survival of 22.1% (95% CI 15.2–29.0; p = 0.001). High serum MMP-14 was a statistically significant prognostic factor among patients with an intestinal type of cancer (hazard ratio [HR] 3.54; 95% CI 1.51–8.33; p = 0.004), but not among patients with a diffuse type. The serum MMP-14 level remained an independent prognostic factor in our multivariate survival analysis (HR 1.55; 95% CI 1.02–2.35; p = 0.040). This study indicates for the first time that high serum soluble MMP-14 levels in gastric cancer serves as a marker for a poor prognosis, possibly indicating the presence of distant metastases.

## Introduction

The incidence of gastric cancer has decreased in the Western world due to changes in lifestyle and decreasing smoking rates, yet survival rates remain modest. For instance, the 5-year survival of patients with advanced disease reached less than 30% [1,2]. Typically, gastric cancer diagnosis occurs at an advanced stage, too late for effective curative treatment. The prognosis could be improved with more precise knowledge of the underlying pathophysiology and earlier diagnosis.

Gastric cancer is heterogeneous by nature and currently classified mainly according to the Tumor-Node-Metastasis (TNM) and the Laurén classifications [3,4]. Classification subgroups typically result in differences in responses to treatment and survival rates, suggesting additional underlying mechanisms [5]. Currently we know of only a few biomarkers associated with gastric cancer, most notably carcinoembryonic antigen (CEA) and carbohydrate antigen 19–9 (CA 19–9), although their clinical usefulness remains debated due to the low sensitivity and specificity rates [5–7]. To detect gastric cancer before a total outbreak of the tumor and to more precisely follow disease progression and prognosis, we need to identify biomarkers with better predictive qualities [8].

Matrix metalloproteinases (MMPs), zinc-containing genetically distinct but structurally related endopeptidases, exhibit diverse biochemical functions, such as the capability to promote cancer cell invasion and the formation of metastases [9]. MMPs promote cancer progression by cleaving and activating structural components of the histological configuration and by modifying immune and defensive reactions [10]. A high MMP expression appears to associate with more aggressive malignant behavior in various types of cancer [11–19].

Among the 26 identified MMPs, matrix metalloproteinase-14 (MMP-14), also known as membrane-type matrix metalloproteinase-1, belongs to the membrane-bound MMPs. In previous studies, a high immunohistochemical MMP-14 expression in tissue samples associated with increased metastasis and a worse prognosis in gastric cancer [17,18,20,21].

Studies of MMP-14 have been primarily centered on tissue samples, although few findings addressing systemic levels of soluble or shedded forms of MMP-14 appear in the literature. In a large-scale study of 810 gastric cancer patients, a high MMP-14 gene expression in peripheral blood and bone marrow strongly associated with distant metastasis [22]. Furthermore, studies on circulating levels of MMP-14 in breast and ovarian cancers concluded that MMP-14 levels appeared elevated in cancer patients compared to controls [23,24]. Finally, MMP-14-expressing gastric cancer cell lines, studied both in mice and in vitro, showed an increased invasion and metastatic spread compared to MMP-14 knockdown versions of the cells [25,26].

To the best of our knowledge, studies of MMP-14 protein levels of gastric cancer patients’ sera remain unpublished. Therefore, this study aims to investigate the value of serum MMP-14 as a biomarker in gastric cancer patients.

## Materials and methods

### Patients

The patient cohort for this study consisted of 240 gastric adenocarcinoma patients who underwent surgery at the Helsinki University Hospital, Finland, between 2000 and 2009. We consecutively included patients undergoing surgery for gastric adenocarcinoma in our study material, excluding individuals with a previous history of malignancies or any synchronous cancers. We compared the gastric cancer patients to a cohort consisting of 48 patients with benign diseases (controls) who underwent surgery between 2000 and 2012.

Among cancer patients, 118 (49.2%) were men with a median age of 67.4 years (interquartile range [IQR] 57.1–76.5). Intestinal tumors were found in 84 (35.0%) patients. Surgery was performed with a curative intent on 182 (75.8%) patients. The median follow-up time was 2.3 years (1 day to 17.2 years), with 55 (22.9%) patients still alive at the end of follow-up. The 5-year disease-specific survival for the cancer patient cohort reached 44.7% (95% CI 41.3–48.1). We used the seventh version of the TNM classification for cancer staging.

Among controls, 27 (56.3%) were men with a median age of 61.0 years (IQR 55.0–71.1). The median follow-up time for controls reached 8.9 years (102 days to 17.8 years), with 26 (54.2%) patients still alive at the end of follow-up. The overall 5-year survival reached 80.9% (95% CI 75.2–86.6). Controls underwent surgery for peptic ulcer disease, benign gastric tumors, duodenal perforation, duodenal polyps, other non-malignant reasons, or gastroscopy to clarify the causes of and diagnose gastro-esophageal reflux disease, hematemesis, or esophageal hiatus hernia.

We updated survival data in November 2017. Information was obtained from patient records, Statistics Finland, and the Population Register Center of Finland.

The Surgical Ethics Committee of Helsinki University Hospital approved this study (Dnro HUS 226/E6/ 06, extension TMK02 §66 17.4.2013), and the National Supervisory Authority of Welfare and Health granted the license to study archived tissue samples without specific individual consent (Valvira Dnro 10041/06.01.03.01/2012).

### Serum samples

Blood samples analyzed were drawn within 30 days prior to surgery with a median of 1 day preoperatively. Serum samples were aliquoted and stored at -80°C until analysis. Serum MMP-14 levels were determined using a commercially available enzyme-linked immunosorbent assay (ELISA) kit, the Human MMP14 ELISA Kit (Abcam, Cambridge, UK), according to the manufacturer’s instructions. Briefly, immediately prior to use, we prepared serially diluted standards with the help of a sample diluent according to the kit’s protocol. Subsequently, 50 μl of all standards and undiluted samples were added to each pre-coated well, as well as 50 μl of the antibody cocktail, following the protocol. After incubating on a plate shaker at room temperature for 1 h, the wells were washed to remove any unbound material. According to the manufacturer instructions, 100-μl substrate was piped into each well and incubated for 10 min. After the 10-min incubation period, the reaction was stopped by adding the stop solution. Detection was carried out by measuring the absorbance at 450 nm using the Victor^TM^ X4 (Wallac Oy, Turku, Finland by PerkinElmer Singapore). Detection limit for serum MMP-14, reported by the manufacturer, was 0.137 ng/ml. All of the solutions used in this procedure were included in the ELISA kit.

### Statistical analyses

We explored statistical differences between the serum MMP-14 levels of the gastric cancer patient group and controls using the Mann-Whitney U-test. In addition, we calculated associations between serum MMP-14 and clinicopathological variables using the Mann-Whitney U-test or the Kruskal-Wallis test. We also constructed the survival figures according to the Kaplan-Meier method and tested for statistically significant differences using the log-rank test. The Cox proportional hazard model was used in order to calculate the hazard ratios for uni- and multivariate survival analyses. The factors entered into the Cox proportional multivariate survival analysis consisted of age, stage, Laurén classification, and the serum MMP-14 level, where stage was processed as a categorical covariate. We found no interaction terms for MMP-14. Each patient’s disease-specific survival time was calculated from the date of surgery until death from gastric cancer. We utilized the receiver operating characteristics (ROC) curve analysis to determine the optimal cut-off point (using the maximum value of Youden’s index) for gastric cancer patients’ serum MMP-14 concentrations, dichotomizing the variable into low (<0.073 ng/ml) and high (≥0.073 ng/ml) groups [27]. The ROC curve analysis was also applied to evaluate the diagnostic value of the serum MMP-14 levels comparing gastric cancer patients to controls. In all analyses, we considered a p-value of less than 0.05 as statistically significant. All analyses were conducted using IBM’s SPSS Statistics, version 24.0 for Mac (IBM Corporation, Armonk, NY, USA).

## Results

### Associations between MMP-14 and clinicopathological variables

We found a positive association between a high serum MMP-14 level and cancer stages III–IV (p = 0.029) and between a high serum MMP-14 level and distant metastasis (p = 0.022; Table 1). We found no other associations between the clinicopathological variables and the serum MMP-14 level.

**Table 1:** Association of serum MMP-14 with clinicopathological variables in 240 gastric cancer patients.

	Serum MMP-14		
	Low (%)	High (%)	p value
**Age, years**			
<67	104 (80.6)	25 (19.4)	0.592^1^
≥67	97 (87.4)	14 (12.6)	
**Gender**			
Male	99 (83.9)	19 (16.1)	0.803^1^
Female	102 (83.6)	20 (16.4)	
**Stage**			
I	44 (89.8)	5 (10.2)	0.029^2^
II	51 (94.4)	3 (5.6)	
III	76 (80.9)	18 (19.1)	
IV	30 (69.8)	13 (30.2)	
**Tumor classification (pT)**			
pT1–2	64 (88.9)	8 (11.1)	0.619^1^
pT3–4	137 (81.5)	31 (18.5)	
**Lymph node metastasis (pN)**			
pN0	66 (88.0)	9 (12.0)	0.858^1^
pN1–3	129 (82.7)	27 (17.3)	
**Distant metastasis (pM)**			
M0	171 (86.8)	26 (13.2)	0.022^1^
M1	30 (69.8)	13 (30.2)	
**Laurén classification**			
Intestinal	75 (89.3)	9 (10.7)	0.143^1^
Diffuse	126 (80.8)	30 (19.2)	

Abbreviations: MMP-14 = Matrix metalloproteinase-14

^1^ Mann-Whitney U-test

^2^ Kruskal-Wallis test

### Survival analyses

**Fig 1:**
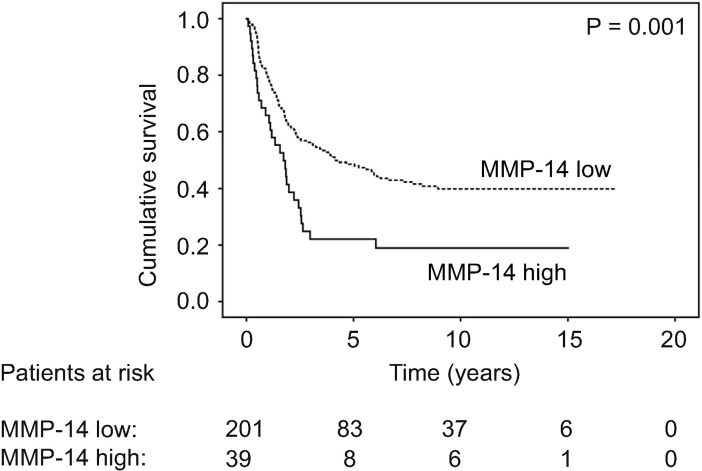
Disease-specific survival of gastric cancer patients according to serum MMP-14 levels. Survival curves were calculated using the Kaplan-Meier method with the p value based on the log-rank test.

Patients with a low serum MMP-14 had a 5-year disease-specific survival of 49.2% (95% CI 45.5–52.9), whereas patients with a high serum MMP-14 had a 5-year survival of 22.1% (95% CI 15.2–29.0; p = 0.001; Fig 1). Serum MMP-14 remained an independent prognostic factor in the multivariate survival analysis (hazard ratio [HR] 1.55; p = 0.040; 95% CI 1.02–2.35; Table 2). Other significant prognostic factors in the multivariate survival analysis consisted of age, stage, and Laurén classification.

**Table 2:** Uni- and multivariate survival analyses for gastric cancer patients.

	Univariate survival analysis			Multivariate survival analysis		
	HR	95% CI	p value	HR	95% CI	p value
**Age, years**						
<67	1.00			1.00		
≥67	1.44	1.08–1.94	0.015	2.52	1.76–3.60	<0.001
**Stage**						
I	1.00			1.00		
II	4.98	2.06–12.1	<0.001	6.88	2.35–20.1	<0.001
III	14.8	6.44–33.9	<0.001	22.0	7.98–60.8	<0.001
IV	41.5	17.7–97.8	<0.001	74.5	25.8–215	<0.001
**Laurén classification**						
Intestinal	1.00			1.00		
Diffuse	1.45	1.06–1.98	0.021	2.23	1.50–3.32	<0.001
**Serum MMP-14**						
Low	1.00			1.00		
High	1.92	1.28–2.88	0.002	1.55	1.02–2.35	0.040

Abbreviations: HR = hazard ratio, CI = confidence interval, MMP-14 = Matrix metalloproteinase-14

**Fig 2:**
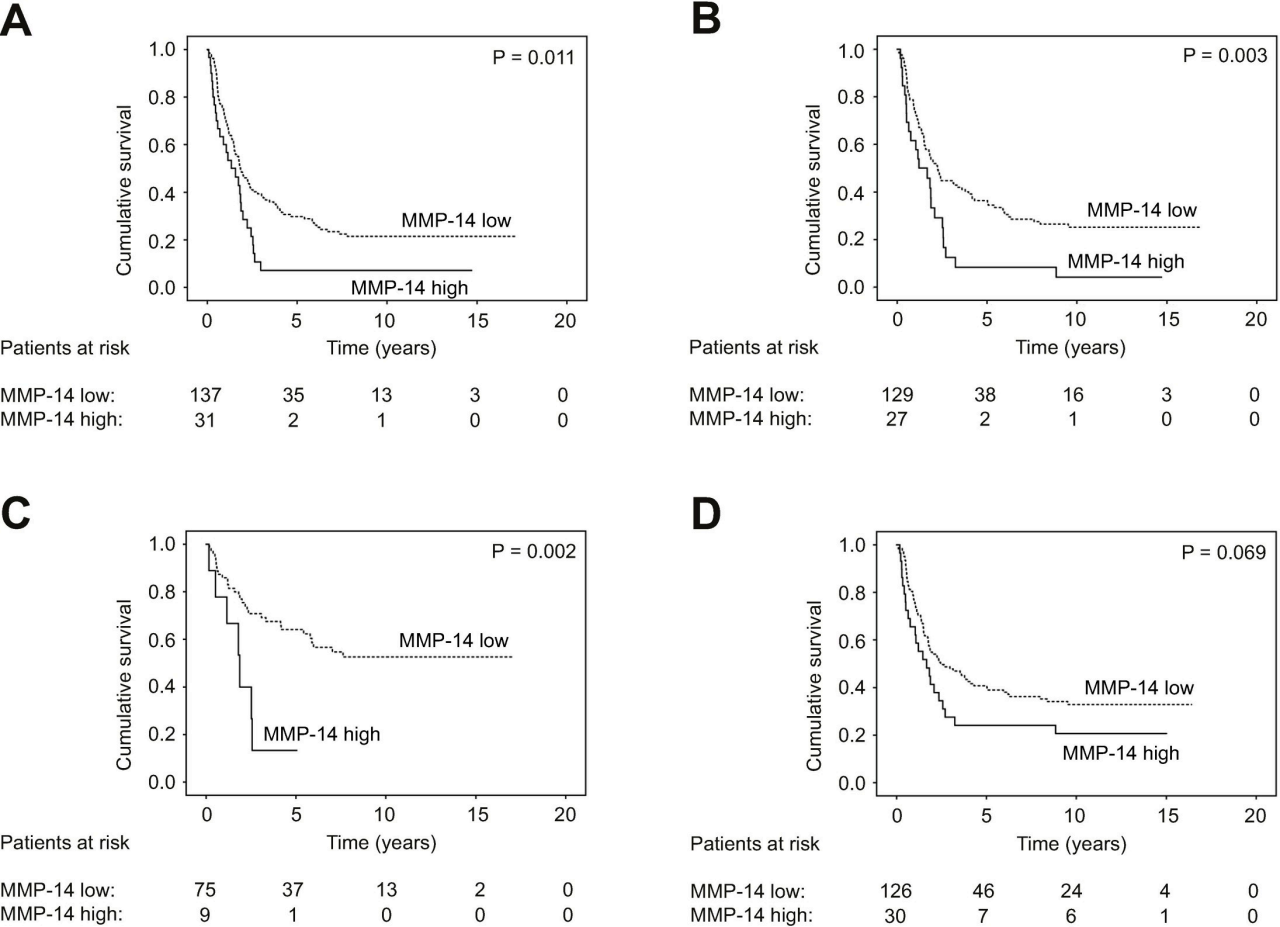
Disease-specific survival in subgroups of gastric cancer patients according to the Kaplan-Meier method. Low versus high serum MMP-14 levels in patients with A) a pT3–4 tumor classification, B) lymph-node metastases, C) an intestinal cancer, and D) a diffuse type of cancer according to the Laurén classification. The p value was calculated using the log-rank test.

Among the subgroups of patients with pT3–4 tumors (HR 1.74; 95% CI 1.13–2.67; p = 0.012; Fig 2A), lymph-node metastases (HR 1.96; 95% CI 1.24–3.09; p = 0.004; Fig 2B), or an intestinal type of cancer (HR 3.54; 95% CI 1.51–8.33; p = 0.004; Fig 2C; Table 3), a high serum MMP-14 served as a prognostic factor. For patients with an intestinal type of cancer, 5-year survival reached 64.1% (95% CI 58.2–70.0) among those with a low serum MMP-14 and 13.3% (95% CI 1.0–25.6; p = 0.002; Fig 2C) among patients with a high serum MMP-14. Among patients with a diffuse type of cancer (HR 1.54; 95% CI 0.96–2.45; p = 0.071; Fig 2D), a high serum MMP-14 did not serve as a prognostic factor. In addition, MMP-14 associated with a worse prognosis among men (HR 2.74; 95% CI 1.54–4.87; p = 0.001; Table 3).

**Table 3:** Univariate survival analysis by subgroups, comparing a high to a low serum MMP-14 value among 240 gastric cancer patients.

	High serum MMP-14		
	HR	95% CI	p value
**Age, years**			
<67	2.02	1.19–3.42	0.009
≥67	2.05	1.07–3.94	0.031
**Gender**			
Male	2.74	1.54–4.87	0.001
Female	1.42	0.79–2.54	0.236
**Stage**			
I	N/A		
II	2.32	0.54–10.0	0.261
III	1.26	0.72–2.20	0.417
IV	1.78	0.88–3.57	0.107
**Tumor classification (pT)**			
pT1–2	2.23	0.62–8.02	0.218
pT3–4	1.74	1.13–2.67	0.012
**Lymph node metastasis (pN)**			
pN0	1.45	0.42–5.01	0.559
pN1–3	1.96	1.24–3.09	0.004
**Distant metastasis (pM)**			
pM0	1.64	0.98–2.74	0.058
pM1	1.78	0.88–3.57	0.107
**Laurén classification**			
Intestinal	3.54	1.51–8.33	0.004
Diffuse	1.54	0.96–2.45	0.071

Abbreviations: MMP-14 = Matrix metalloproteinase-14, HR = hazard ratio, CI = confidence interval, N/A = not available

### Diagnostic value of serum MMP-14

Serum MMP-14 levels were higher among controls than among gastric cancer patients (p = 0.002; S1 Table). Among gastric cancer patients, the serum MMP-14 level was high in only 39 (16.3%) patients. Among controls, serum MMP-14 was high in 14 (29.2%) patients. The area under the curve in the ROC analysis was 0.392 (95% CI 0.30–0.48; p = 0.019), suggesting a low diagnostic value. The sensitivity of a high serum MMP-14 level to detect gastric cancer reached 16.3%, while the specificity was 70.8%.

## Discussion

Among our cohort of 240 gastric cancer patients we found that serum MMP-14 served as an independent prognostic factor. To our knowledge, this represents the first study on serum levels of soluble MMP-14 in gastric cancer. We also found that survival was worse among patients with a high serum MMP-14, particularly among men, patients with pT3–4 tumors, in the presence of lymph-node metastases, or accompanying an intestinal cancer. Furthermore, a high serum MMP-14 level associated with metastatic disease. Our results agree with MMP-14’s previously known role in promoting cancer cell invasion and the formation of metastases [9]. However, in our study, serum MMP-14 did not serve as a diagnostic tumor marker.

Only a few studies of MMP-14 in gastric cancer exist and none examined serum levels. The high MMP-14 immunoexpression in 205, 225, 184, and 96 gastric cancer tissues, and in a meta-analysis of 594 patients, served as a prognostic factor and associated with age, stage, tumor grade, lymph-node metastases, distant metastases, and tumor invasion [11,17,20,21]. Our findings regarding MMP-14 in the serum of gastric cancer patients agree with these studies.

Mimori et al. [22] studied the MMP-14 levels of peripheral blood and bone marrow among 810 gastric cancer patients. They found that MMP-14 gene expression, determined using RT-PCR, strongly associated with metastatic disease. Similarly, in our study, high MMP-14 serum levels associated with metastatic disease.

The MMP-14 protein and gene expression in gastric cancer tissue appear elevated in gastric cancer patients when compared to controls [17,18,21]. In addition, in a study of 18 breast cancer patients and 11 healthy controls, the serum MMP-14 levels determined using an enzyme-linked immunosorbent assay were significantly higher in cancer patients than in controls [23]. In other studies of MMP-14 serum levels specifically among breast and ovarian cancer patients, circulating MMP-14 levels were higher in comparison with healthy individuals [23,24]. In contrast, in our study, serum MMP-14 did not serve as a diagnostic marker for gastric cancer, since the serum levels detected among controls were higher than those among our patients. This suggests that in our study the high MMP-14 levels in the serum from patients with benign disease may originate from cells other than the gastric mucosa. Additionally, the serum MMP-14 levels among controls and cancer patients may be affected by an uneven distribution and shedding of MMP-14 from healthy and malignant cell membranes. Due to the significant membrane-binding properties of MMP-14, its serum levels do not necessarily directly reflect its overall expression in malignant disease, including in gastric cancer.

Serum MMP-14 served as a strong prognostic factor in patients with an intestinal cancer. Among patients with a diffuse type of cancer, serum MMP-14 did not serve as a prognostic factor, nor did serum MMP-14 associate with the Laurén classification. Nevertheless, this result is of high interest, since intestinal and diffuse types of cancer behave differently and may be considered distinct diseases. Because patients with an intestinal cancer generally have a better prognosis, finding a marker to identify patients with a poor prognosis may prove beneficial.

MMP-14 has been studied in laboratory conditions, resulting in findings that agree with ours. For example, Zheng et al. [25] studied the capability of MMP-14 downregulation to prevent carcinogenic features of gastric cancer cells. They found that MMP-14 suppression resulted in reduced migration, invasion, and angiogenesis in a time- and dose-dependent fashion. In comparison, Nonaka et al. [26] studied the tumor-promoting function of MMP-14 in mice, whereby the suppression of MMP-14 lead to a diminished malignant behavior.

MMP-14 has been suggested as a novel target for therapy. Thus far, however, no efficient inhibitor for MMP-14 for clinical use exists [28]. Liu et al. [29] studied the effect of a synthetic broad-spectrum MMP inhibitor (MMPI), prinomastat, in an orthotopic lung cancer model in which tumors express MMP-14, MMP-2, and tissue inhibitor of metalloproteinases-2 (TIMP-2). Prinomastat was administered as a single-therapy agent and in combination with carboplatin, resulting in a reduction of kidney metastases, while in combination with carboplatin survival improved. Consequently, studies of prinomastat have proceeded to clinical trials [30].

The strengths of our study include the relatively large patient cohort with reliable follow-up information and our validation of previous findings. The single-center setting introduces a bias, since it decreases the generalizability of our findings. Further research on this topic from additional large, well-defined patient cohorts remain necessary in order to confirm our findings. Due to the retrospective study design, accessing patient details regarding other well-known risk factors, such as lymphatic emboli, venous invasion, perineural invasion, and tumor subsite, is difficult and potentially contains inaccuracies. Thus, we have not included them in the analyses.

To conclude, this study suggests for the first time that high serum levels of soluble MMP-14 in gastric cancer can serve as a marker for a worse prognosis and may indicate the presence of distant metastases.

## Supporting information

S1 Fig ROC curve for gastric cancer patients. We used the ROC curve to determine the optimal cut-off value for gastric cancer patients’ serum MMP-14 levels.
(TIF)

S1 Table Serum MMP-14 in gastric cancer patients versus controls. (XLSX)
