# Supplementary figures and images for: High serum MMP-14 predicts worse survival in gastric cancer
Source: PLoS One. 2018 Dec 7;13(12):e0208800. doi: 10.1371/journal.pone.0208800 (PMC6285995; doi:10.1371/journal.pone.0208800)

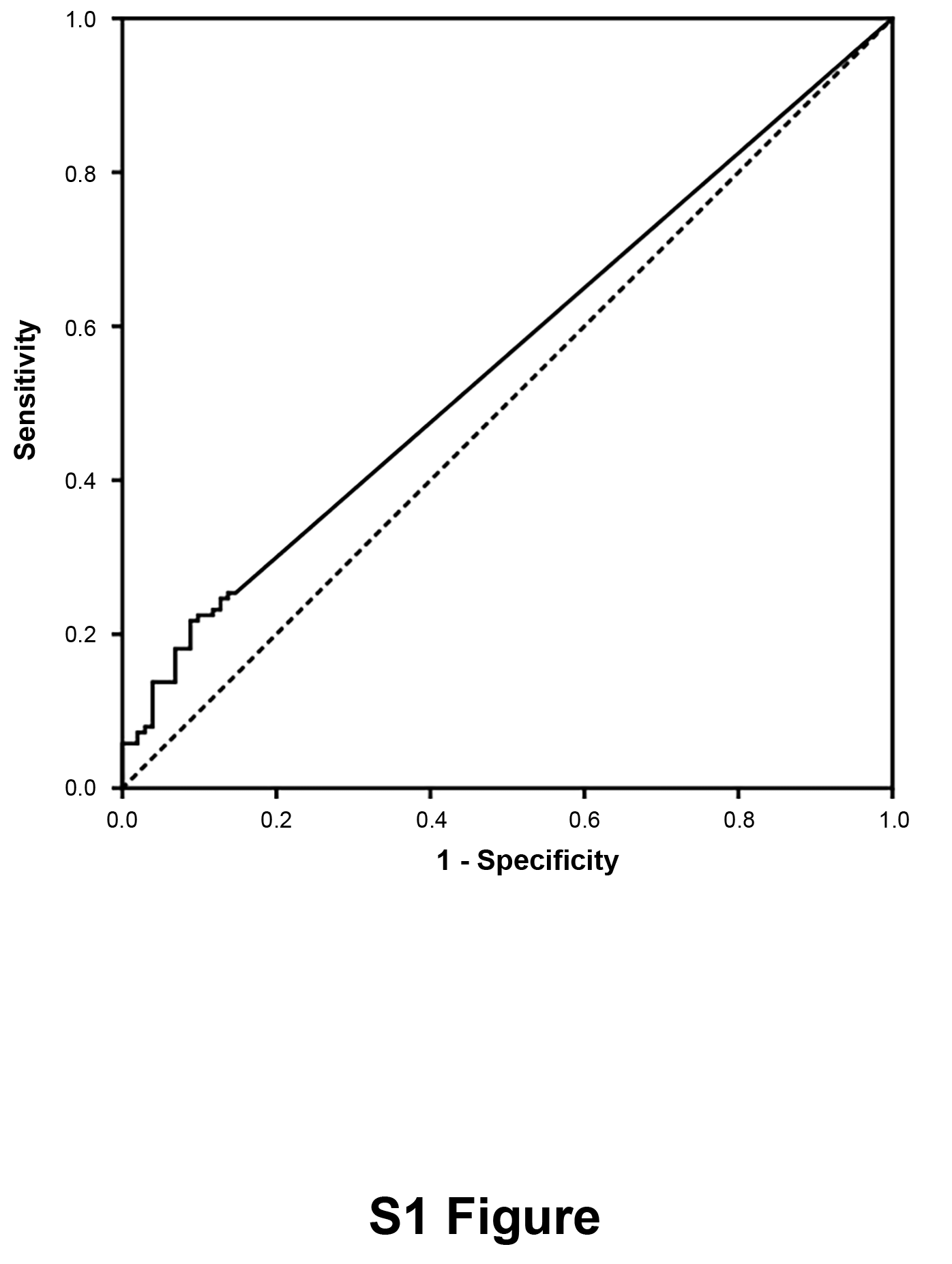

Supplement: S1 Fig — We used the ROC curve to determine the optimal cut-off value for gastric cancer patients’ serum MMP-14 levels. (TIF) [file pone.0208800.s001.tif]
